# Characterization of long non-coding RNA and messenger RNA profiles in laryngeal cancer by weighted gene co-expression network analysis

**DOI:** 10.18632/aging.102419

**Published:** 2019-11-18

**Authors:** Huanhuan Liu, Yi Sun, Huan Tian, Xiaolian Xiao, Jiaqi Zhang, Yongzhen Wang, Fengyan Yu

**Affiliations:** 1 Department of Plastic Surgery, Sun Yat-sen Memorial Hospital, Sun Yat-sen University, Guangzhou, Guangdong, China; 2 Department of Breast Surgery, Sun Yat-sen Memorial Hospital, Sun Yat-sen University, Guangzhou, Guangdong, China; 3 Guangdong Provincial Key Laboratory of Malignant Tumor Epigenetics and Gene Regulation, Sun Yat-sen Memorial Hospital, Sun Yat-sen University, Guangzhou, Guangdong, China

**Keywords:** biomarker, lncRNA, mRNA, laryngeal cancer, WGCNA

## Abstract

Laryngeal cancer (LC) is a malignant tumor in the head and neck region. It was recently elucidated that long non-coding RNAs (lncRNAs) participate in the pathogenesis of LC. However, the detailed mechanism of lncRNA in LC and whether long non-coding RNAs serve as effective biomarkers remains unclear. Ribonucleic acid (RNA) sequence data of LC and 11 patient clinical traits were extracted from The Cancer Genome Atlas (TCGA) database and analyzed by weighted gene co-expression network analysis (WGCNA). A total of 9 co-expression modules were identified. The co-expression Pink module significantly correlated with four clinical traits, including history of smoking, lymph node count, tumor status, and the success of follow-up treatment. Based on the co-expression Pink module, lncRNA-microRNA (miRNA)-messenger RNA (mRNA) and lncRNA-RNA binding protein-mRNA networks were constructed. We found that 8 lncRNAs significantly impacted overall survival (OS) in LC patients. These identified lncRNA and hub gene biomarkers were also validated in multiple LC cells *in vitro* via qPCR. Taken together, this study provided the framework of co-expression gene modules of LC and identified some important biomarkers in LC development and disease progression.

## INTRODUCTION

As one of the most common neoplasms of the neck and head region, prognosis of laryngeal carcinoma (LC) remains poor. The incidence of LC is increasing gradually, and presents an early-rising trend. The patient data from the Surveillance, Epidemiology, and End Results database (SEER) from 2004 to 2015 showed that when patients were diagnosed with LC at a younger age (< 40 years old) the disease tended to be more aggressive and associated with a poorer survival rate than in older patients (> 40 years old) [1]. Although patients diagnosed at an early stage could be treated surgically following combination therapy [2], most cases were diagnosed at an advanced stage due to lack of early diagnostic capability. These patients suffered from symptoms such as persistent cough, stridor, bad breath, earache and difficulty swallowing. There are very limited therapeutic methods available for advanced patients who could not be treated surgically for whatever reason [3]. Thus, there is an urgent need to discover new diagnostic biomarkers specifically targeting LC for the purpose of early diagnosis and development of new treatments which could improve survival rate and quality of life for LC patients.

Long noncoding RNAs (lncRNAs) are clusters of noncoding transcripts longer than 200 nucleotides which play key roles in various biological processes involving transcriptional regulation, chromatin modification, RNA editing, posttranscriptional processing, molecules metabolism, cell cycle regulation, alternative splicing, immune response, and organelle biogenesis [4–7]. Many previous studies have confirmed that lncRNAs are closely associated with proliferation, metastasis, drug sensitivity, and progression of the tumor [8]. Recent studies demonstrate that RNA species could regulate each other by competing for shared miRNA response elements. This regulatory model is called competing endogenous RNA (ceRNA). The identified ceRNAs include lncRNAs, which provide a new perspective of regulatory mechanism of lncRNA [9, 10]. Additionally, lncRNA can also combine with other RNA binding proteins, such as transcription factor, to affect target mRNA expression [11]. LncRNA has two main functions in transcription factors and other transcriptional regulatory proteins, including recruitment and inhibition. LncRNA may promote or inhibit the transcription process in specific conditions [12]. Weighted Gene Co-expression Network Analysis (WGCNA) is used to find highly correlated gene clusters (co-expression modules) and widely used to demonstrate correlation between gene-based networks and clinical phenotypes based on microarray data or RNA sequencing data [13]. The identified co-expression modules can be summarized using the module eigengene or intramodular hub genes. Correlation networks of genes and clinical phenotypes can be used to identify potential biomarkers or therapeutic targets [14]. These methods are increasingly being applied in various biological contexts, such as cancer research, proteomic data, metabolomics data, and analysis of imaging data [15]. For example, RNA sequence data of uveal melanoma and patient clinic traits was obtained from TCGA database. Co-expression modules were built by WGCNA and applied to investigate the relationship underlying modules and clinic traits. Their findings demonstrated that hub genes SLC17A7, NTRK2, ABTB1 and ADPRHL1 might play a vital role in recurrence of uveal melanoma [16]. Another study also which used WGCNA to identify 6 modules associated with pathological stage and grade discovered that the co-expression Blue module was the most relevant module in clear cell renal cell carcinoma [17]. Their findings showed that 9 genes were associated with progression and prognosis of renal clear cell carcinoma patients including PTTG1, RRM2, TOP2A, UHRF1, CEP55, BIRC5, UBE2C, FOXM1 and CDC20. Currently, there is only one study which uses WGCNA to decipher potential hub genes driving the development of LC [18].

The present study collected RNA sequencing data (including lncRNA expression data and mRNA expression data) from The Cancer Genome Atlas (TCGA) database to elucidate significant co-expression modules in LC patients compared to healthy controls. Those modules were closely related to clinical traits in LC patients, and the genes in those identified modules might affect the development and progression of LC. The co-expression Pink module was selected for further analysis because it contains many significant clinical traits and could be developed into novel biomarkers for LC patients. Furthermore, analysis of lncRNA-miRNA-mRNA and lncRNA-RNA binding protein-mRNA networks might offer new insight into the molecular mechanisms of LC, which could be helpful in improving early diagnosis and overall prognosis for LC patients.

## RESULTS

### Construction of co-expression modules of LC

**Figure 1:**
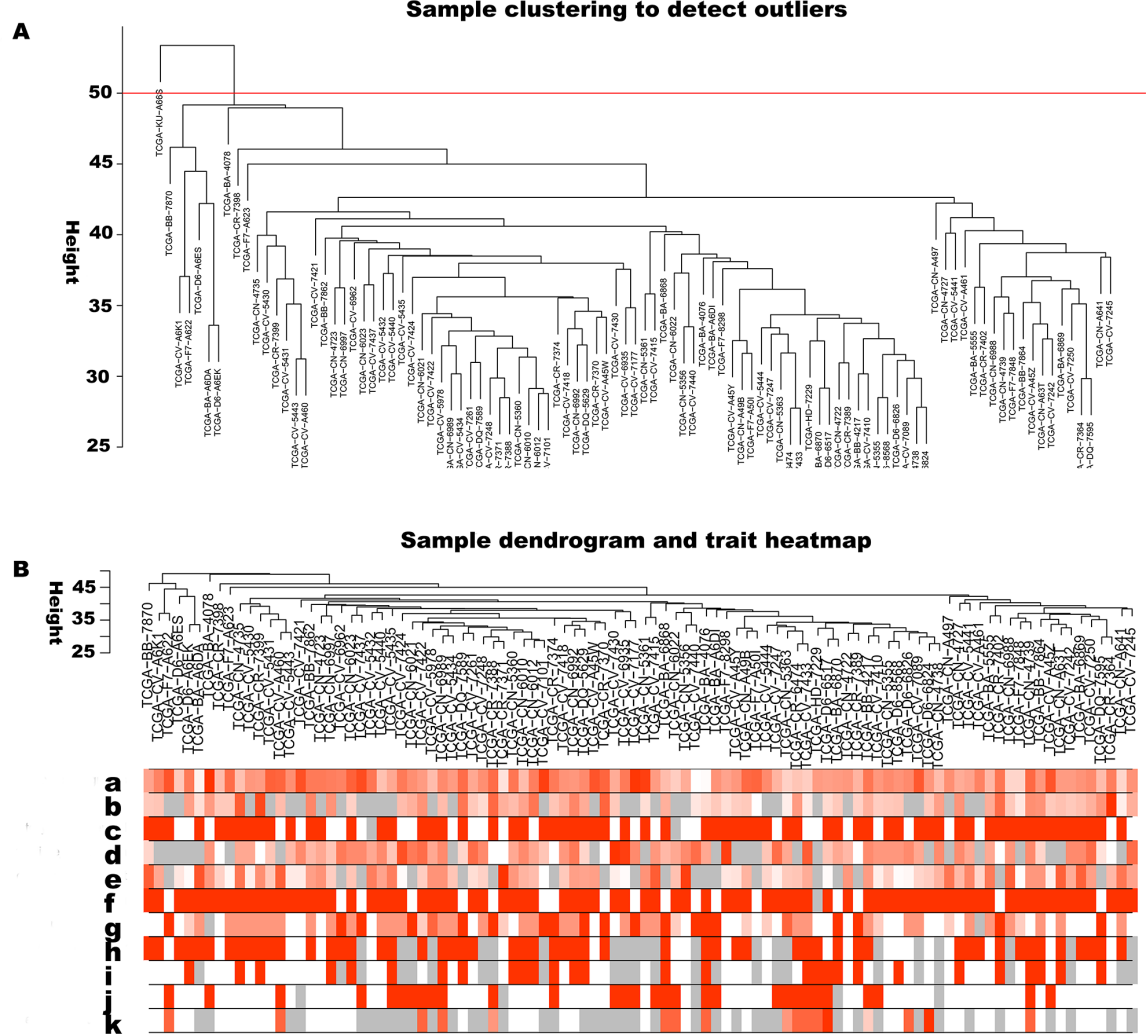
**Sample cluster analysis based on RNA data from TCGA database.** (**A**) Sample clustering to detect outliers based on RNA data. The red line represents the cut-off of data filtering in the step of data preprocessing. (**B**) Sample dendrogram and trait heatmap based on gene expression data and clinical data. (**a**) age at initial pathologic diagnosis, (**b**) history of smoking, (**c**) history of alcohol consumption, (**d**) intermediate dimension, (**e**) lymph node count, (**f**) neck lymph node dissection, (**g**) pathologic N stage, (**h**) radiation therapy, (**i**) targeted molecular therapy, (**j**) tumor status, (**k**) success of follow-up treatment.

**Figure 2:**
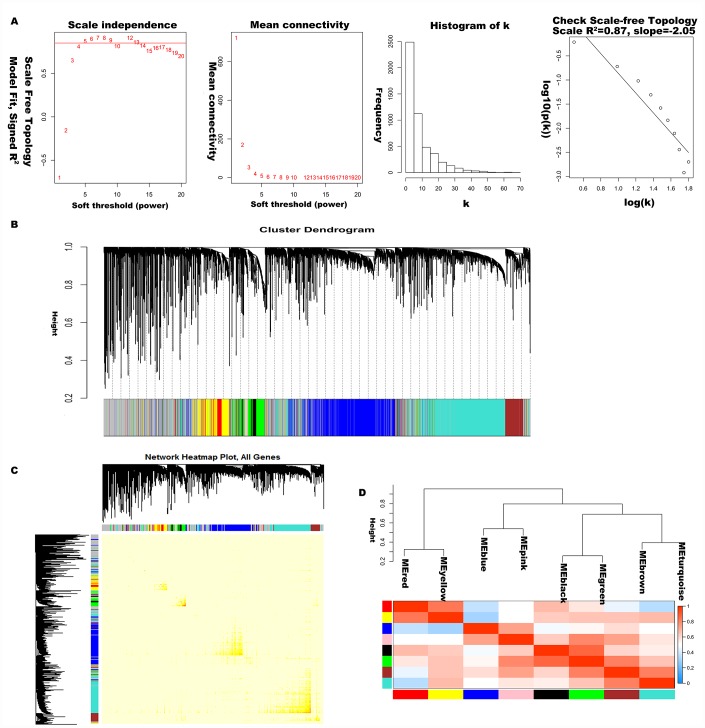
**Construction of co-expression modules of LC.** (**A**) Analysis of network topology for various soft-threshold powers. Check scale-free topology, and here the adjacency matrix was defined using soft-thresholds with beta= 5. (**B**) Clustering dendrograms of genes, with dissimilarity based on topological overlap, together with assigned module colors. (**C**) The heatmap depicts the topological overlap matrix (TOM) among genes based on co-expression modules. (**D**) Visualizing the gene network using a heatmap plot.

The 5000 genes (including 2500 lncRNA and 2500 mRNA expression data) were normalized by Limma package with Voom function. The auxiliary data was then removed and the expression data was transposed for further WGCNA analysis. The expression values of top 2500 lncRNAs in 99 LC samples (Supplementary Table 1) and top 2500 mRNAs in 99 LC samples (Supplementary Table 2) were used to develop co-expression modules with WGCNA package. The clinical characteristics information of these LC patients is listed in Supplementary Table 3, including age at initial pathologic diagnosis, history of smoking, history of alcohol consumption, intermediate dimension of tumor, lymph node count, neck lymph node dissection, pathologic N stage, radiation therapy, targeted molecular therapy, tumor status, success of follow-up treatment, and overall survival. The red line (cut height = 50) was the filter which we used to remove outlier samples in sampleTree. The TCGA-KU-A66S sample was excluded after removing outliers in the sample based on gene expression data (Figure 1A). The sample dendrogram and trait heatmap grouped the selected samples into the different clusters, and provided the distribution map of clinical trait data (Figure 1B). The independence and the average connectivity degree of the co-expression modules were decided by power value (β) and scale R^2^ value. First, a set of soft-thresholding powers were plotted (Supplementary Table 4). When the power value was equal to 5, the scale R^2^ was up to 0.87 (Figure 2A). Therefore, we define the adjacency matrix using soft thresholding with beta=5 to construct and identify distinct co-expression gene modules in LC samples. A cluster dendrogram of all selected genes was constructed based on a TOM-based dissimilarity measure. These identified co-expression modules were distributed in different colors (Figure 2B). The interactions of these co-expression modules were analyzed with the Pearson correlation coefficient (Figure 2C). The darker background indicated higher module correlation. Hierarchical clustering of module eigengenes summarizing the modules were found in the clustering analysis. Branches of the dendrogram (the meta-modules) were grouped together based on the correlation of eigengenes. Each row and column in the heatmap plot of topological overlap corresponded to one module eigengene labeled by a different color. Each module contains different gene clusters. In the heatmap, blue represented negative correlation, while red represented positive correlation. Squares of red along the diagonal were the meta-modules (Figure 2D).

### Gene co-expression modules correspond to clinical traits

**Figure 3:**
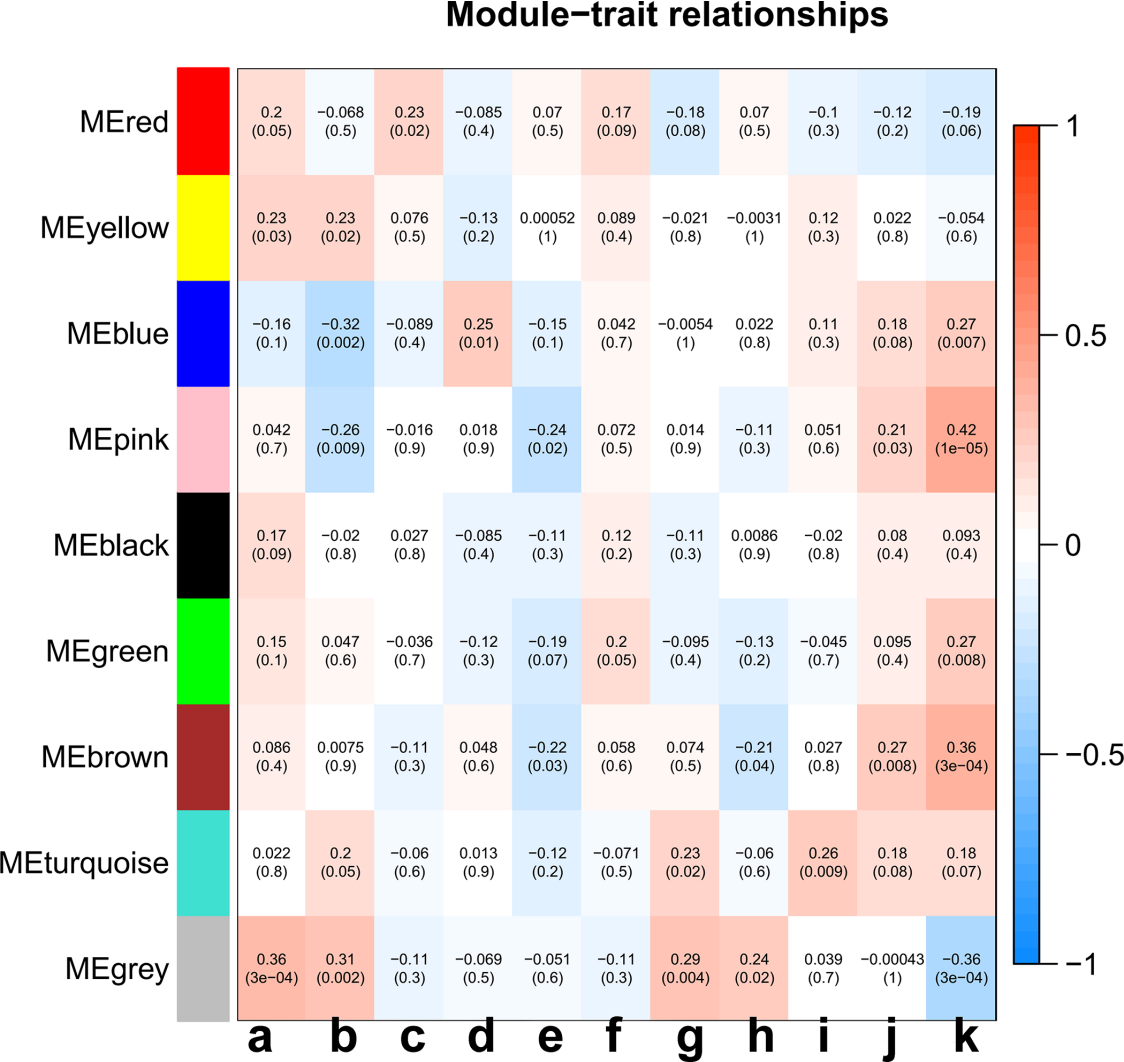
**Analysis of module-trait relationships of LC based on TCGA data.** Each row corresponds to a module eigengene, and column to a trait. (**a**) age at initial pathologic diagnosis, (**b**) history of smoking, (**c**) history of alcohol consumption, (**d**) intermediate dimension, (**e**) lymph node count, (**f**) neck lymph node dissection, (**g**) pathologic N stage, (**h**) radiation therapy, (**i**) targeted molecular therapy, (**j**) tumor status, (**k**) success of follow-up treatment.

**Figure 4:**
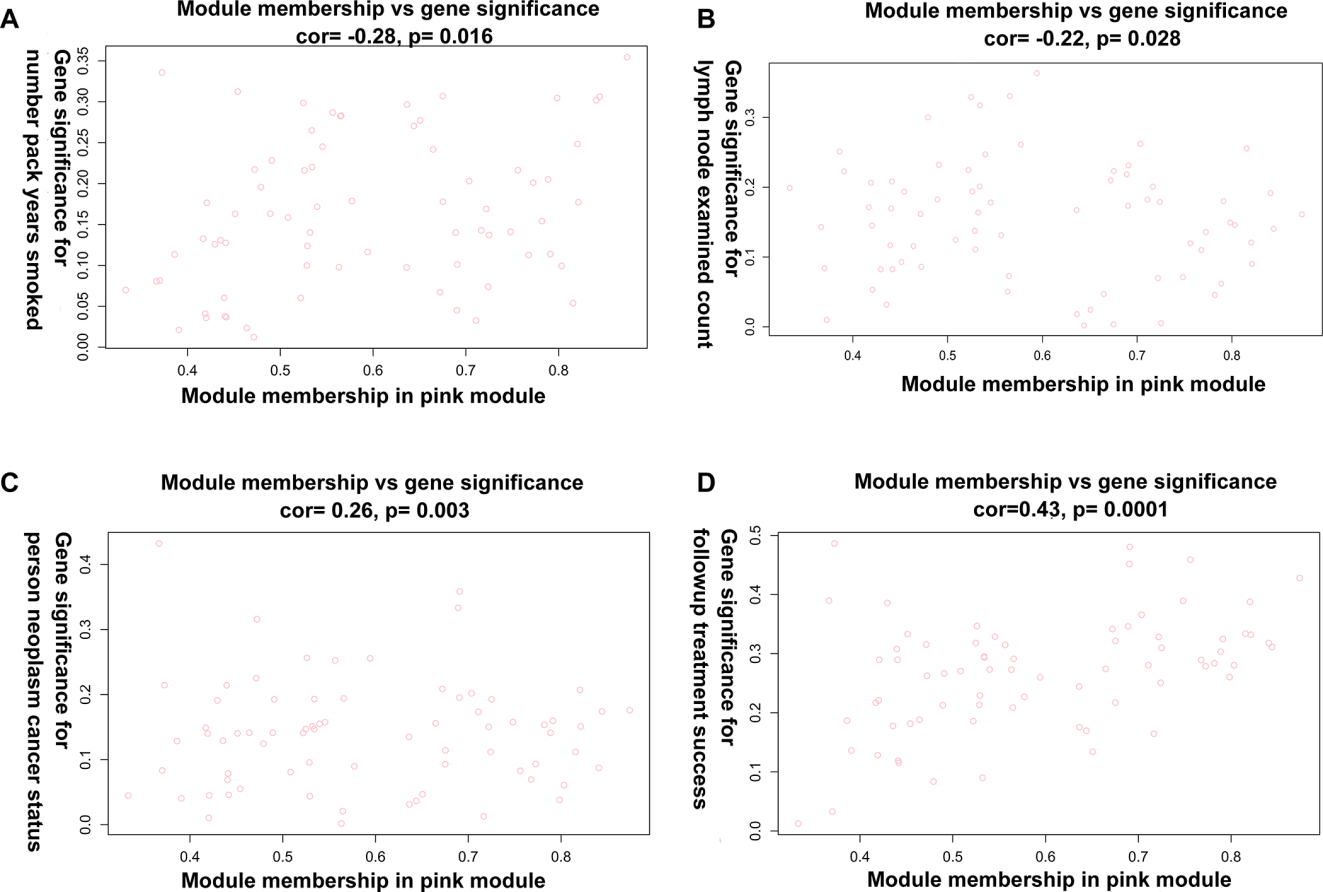
**A scatterplot of Gene Significance (GS) vs. Module Membership (MM) in the co-expression Pink module.** (**A**) A scatterplot of Gene Significance (GS) for number of packs per year vs. Module Membership (MM). (**B**) A scatterplot of Gene Significance (GS) for lymph node count vs. Module Membership (MM). (**C**) A scatterplot of Gene Significance (GS) for tumor status vs. Module Membership (MM). (**D**) A scatterplot of Gene Significance (GS) for success of follow-up treatment vs. Module Membership (MM).

Principal component analysis of each module elected the first principal component as its eigengenes. Eigengene external traits (including age at initial pathologic diagnosis, history of smoking, history of alcohol consumption, intermediate dimension, lymph node count, neck lymph node dissection, pathologic N stage, radiation therapy, targeted molecular therapy, tumor status, success of follow-up treatment) were correlated with different co-expression modules and the most significant associations were identified (Figure 3). A heatmap of the correlation between module eigengenes and clinical traits of LC showed correlation coefficient (R) and significant difference (*p* value). The Module-trait relationships plot demonstrated that the co-expression Red module contained 121 genes, co-expression Yellow module contained 336 genes, co-expression Blue module contained 1123 genes, co-expression Pink module contained 73 genes, co-expression Black module contained 88 genes, co-expression Green module contained 301 genes, co-expression Brown module contained 372 genes, co-expression Turquoise module contained 1186 genes, and co-expression Grey module contained 1390 genes (Table 1). The hub genes of each module might be potential biomarkers for the specific clinical characteristics. The correlation analysis of gene co-expression module and clinical traits demonstrated that the co-expression Red module was significantly associated with documented alcohol history (R =0.23, p=0.02); the co-expression Yellow module was significantly associated with age at initial pathologic diagnosis(R =0.23, p=0.03), and the number of packs of cigarettes per year the patient had smoked (R=0.23, p=0.02); the co-expression Blue module was significantly associated with intermediate dimension of tumor (0.2 to 1.4 cm) (R=0.25, p=0.01), and success of follow-up treatment (R=0.27, p=0.007); the co-expression Green module was significantly associated with success of follow-up treatment (R=0.27, p=0.008); the co-expression Brown module was significantly associated with tumor status (R=0.27, p=0.008), and success of follow-up treatment (R=0.36, p=3e-04); the co-expression Turquoise module was significantly associated with pathologic N stage (R=0.27, p=0.008), and targeted molecular therapy (R=0.36, p=3e-04); and the co-expression Pink module was significantly associated with cigarette packs per year (R=-0.26, p=0.009), lymph node examined count (R=-0.24, p=0.02), tumor status (R=0.21, p=0.03), and success of follow-up treatment (R=0.42, p=1e-05). A scatterplot of gene significance (y-axis) vs. module membership (x-axis) was shown in the most significant module. Most interestingly, the scatterplot of gene significance (GS) vs. module membership (MM) was plotted in the co-expression Pink module. In modules related to a trait of interest, genes with high module membership often had high gene significance, implying that hub genes of the co-expression Pink module tend to be highly correlated with selected clinical characteristics. The results consistently revealed that MM in the Pink module significantly correlated with history of smoking (R=-0.28, p=0.016), lymph node count (R=-0.219, p=0.028), tumor status (R=0.263, p=0.003), and success of follow-up treatment (R=0.43, p=0.00015) (Figure 4A–4D). The correlation results in the co-expression Pink module showed consistency between module-trait relationships plot and the scatterplot of gene significance (GS) vs. module membership (MM) plot. Considering the correlation coefficient, p value, and consistency between module-trait relationships plot and the scatterplot, we chose the co-expression Pink module for further analysis.

**Table 1:** Number of genes in 9 co-expression modules.

**Module colors**	**Gene frequency**
**red**	121
**yellow**	336
**blue**	1123
**pink**	73
**black**	88
**green**	301
**brown**	372
**turquoise**	1186
**grey**	1390

### Functional enrichment gene analysis in the co-expression Pink module

**Figure 5:**
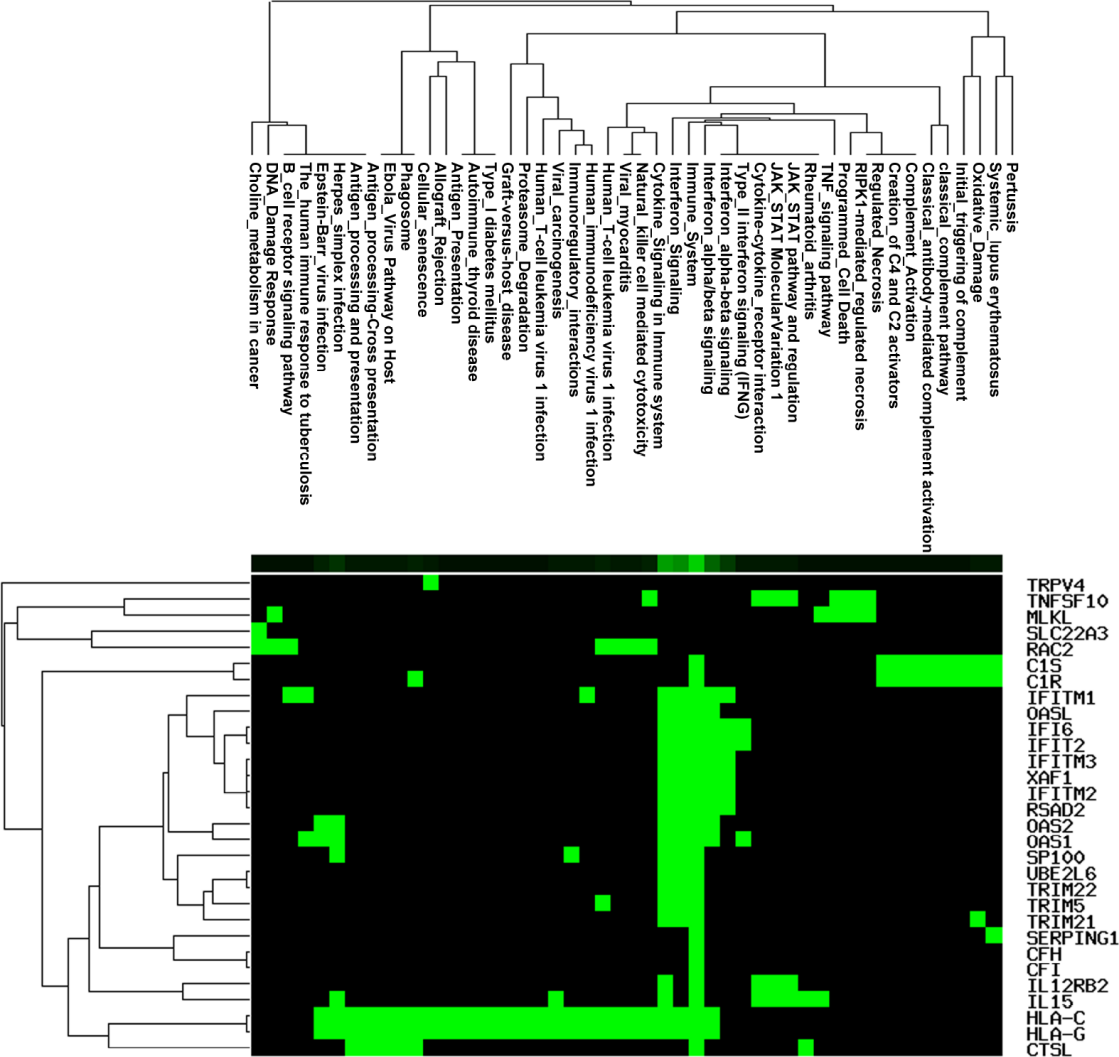
**Pathway enrichment analysis involved in the co-expression Pink module.**

Based on the heatmap, we extracted all 73 of the co-expression Pink module genes (Supplementary Table 5) and hub genes (including IFIT2, XAF1, UBE2L6, IFITM3, HLA-C, CTSL, ARHGDIB, LGALS3BP, IFITM1, MLKL, SERPING1, TRIM21) (Supplementary Table 6). A total of 10 statistically significant signaling pathways were obtained by pathway analysis based on the co-expression Pink module (Table 2). The data revealed 10 distinct clusters which might possibly indicate that LC is potentially related to multiple signaling pathways (Figure 5). Cluster 1 contains cytokine signaling in the immune system, interferon signaling, immune system, interferon alpha/beta signaling, interferon alpha-beta signaling, and type II interferon signaling. Cluster 2 contains interferon gamma signaling, the herpes simplex infection pathway, and the Epstein-Barr virus infection pathway. Cluster 3 contains an endosomal/vacuolar pathway, antigen processing and presentation, antigen processing cross presentation, phagosome, and Ebola Virus pathway. Cluster 4 contains natural killer cell which mediate cytotoxicity, viral myocarditis, human cytomegalovirus infection, and human immunodeficiency virus 1 infection. Cluster 5 contains classical complement pathway, classical antibody-mediated complement activation, pertussis, complement activation, oxidative damage, systemic lupus erythematosus, initial triggering of complement, and creation of C4 and C2 activators. Cluster 6 contains regulated necrosis, RIPK1-mediated regulated necrosis, DNA damage response, and programmed cell death. Cluster 7 contains the human immune response to tuberculosis, B cell receptor signaling pathway, choline metabolism in cancer. Cluster 8 contains antigen presentation, autoimmune thyroid disease, allograft rejection, Graft-versus-host disease, Type-I diabetes mellitus, proteasome degradation, cellular senescence, viral carcinogenesis, human T-cell leukemia virus 1 infection, immunoregulatory interactions between a lymphoid and a non-lymphoid cell, and allograft rejection. Cluster 9 contains JAK-STAT molecular variation 1, and cytokine-cytokine receptor interaction. Cluster 10 contains rheumatoid arthritis and TNF signaling pathway. Most enriched genes of those identified pathways were cytokines, chemokines, histocompatibility antigens, complement factors, autoantigens and/or innate immune response molecules, all of which were thought to play a role in tumorigenesis, immunity, and gene regulation. In recent years, new molecules have been widely reported which are associated with cancer progression and immune escape and there are also strong correlations between antitumoral and immunomodulation effect [19]. The mechanisms that support evolution of the inflammatory environment and its relationship with neoplasms are unclear [20]. Tumor-associated inflammation is predictive of poor prognosis and associated with a variety of tumorigenic phenotypes, including angiogenesis, apoptosis, tumor proliferation and survival, autophagy, invasiveness, and metastasis [21]. Here, we identified an inflammation activation related pathway in the co-expression Pink module. The present understanding of this area is not yet comprehensive, but certain inflammatory pathways have emerged as important mediators of the crosstalk between tumor biology behavior and inflammation in tumors [22].

**Table 2:** The pathways enriched in the pink coexpression module.

**Pathway**	**p Value**	**Benjamini hochberg p value**	**Gene list**
**cluster1**			
Cytokine Signaling in Immune system	7.19E-57	3.16E-55	HLA C;HLA G;IFI6;IFIT2;IFITM1;IFITM2;IFITM3; IL12RB2;IL15;OAS1;OAS2;OASL;RSAD2;SP100;TRIM21; TRIM22;TRIM5;UBE2L6;XAF1
Interferon Signaling	3.98E-24	1.16E-22	HLA C;HLA G;IFI6;IFIT2;IFITM1;IFITM2;IFITM3;OAS1; OAS2;OASL;RSAD2;SP100;TRIM21;TRIM22;TRIM5;UBE2L6; XAF1
Immune System	1.73E-21	3.81E-20	C1R;C1S;CFH;CFI;CTSL;HLA C;HLA G;IFI6;IFIT2;IFITM1; IFITM2;IFITM3;IL12RB2;IL15;OAS1;OAS2;OASL;RSAD2; SERPING1;SP100;TRIM21;TRIM22;TRIM5;UBE2L6;XAF1
Interferon alpha/beta signaling	1.58E-19	2.78E-18	HLA C;HLA G;IFI6;IFIT2;IFITM1;IFITM2;IFITM3;OAS1; OAS2;OASL;RSAD2;XAF1
Interferon alpha beta signaling	5.93E-14	8.70E-13	IFI6;IFIT2;IFITM1;IFITM2;IFITM3;RSAD2;XAF1
Type II interferon signaling (IFNG)	1.29E-04	7.08E-04	IFI6;IFIT2;OAS1
**cluster2**			
Interferon gamma signaling		0.00E+00	HLA C;HLA G;OAS1;OAS2;OASL;SP100;TRIM21;TRIM22; TRIM5
Herpes simplex infection	8.59E-06	6.88E-05	HLA C;HLA G;IL15;OAS1;OAS2;SP100
Epstein Barr virus infection	1.96E-03	5.40E-03	HLA C;HLA G;OAS1;OAS2
**cluster3**			
Endosomal/Vacuolar pathway	4.95E-06	4.36E-05	CTSL;HLA C;HLA G
Antigen processing and presentation	1.13E-03	3.67E-03	CTSL;HLA C;HLA G
Antigen processing Cross presentation	2.99E-04	1.55E-03	CTSL;HLA C;HLA G
Phagosome	6.98E-04	2.79E-03	C1R;CTSL;HLA C;HLA G
Ebola Virus Pathway on Host	5.00E-03	1.22E-02	CTSL;HLA C;HLA G
**cluster4**			
Natural killer cell mediated cytotoxicity	3.98E-04	1.95E-03	HLA C;HLA G;RAC2;TNFSF10
Viral myocarditis	5.17E-04	2.40E-03	HLA C;HLA G;RAC2
Human cytomegalovirus infection	2.20E-02	3.66E-02	HLA C;HLA G;RAC2
Human immunodeficiency virus 1 infection	2.38E-03	6.17E-03	HLA C;HLA G;RAC2;TRIM5
**cluster5**			
classical complement pathway	6.22E-04	2.61E-03	C1R;C1S
Classical antibody mediated complement activation	2.36E-02	3.85E-02	C1R;C1S
Pertussis	1.08E-03	3.67E-03	C1R;C1S;SERPING1
Complement Activation	1.56E-03	4.73E-03	C1R;C1S
Oxidative Damage	5.11E-03	1.21E-02	C1R;C1S
Systemic lupus erythematosus	5.33E-03	1.20E-02	C1R;C1S;TRIM21
Initial triggering of complement	3.26E-02	4.79E-02	C1R;C1S
Creation of C4 and C2 activators	2.77E-02	4.28E-02	C1R;C1S
**cluster6**			
Regulated Necrosis	8.18E-04	3.13E-03	MLKL;TNFSF10
RIPK1 mediated regulated necrosis	8.18E-04	3.00E-03	MLKL;TNFSF10
DNA Damage Response (only ATM dependent)	3.49E-02	4.96E-02	MLKL;RAC2
Programmed Cell Death	4.15E-02	5.80E-02	MLKL;TNFSF10
**cluster7**			
The human immune response to tuberculosis	1.56E-03	4.90E-03	IFITM1;OAS1
B cell receptor signaling pathway	1.54E-02	2.77E-02	IFITM1;RAC2
Choline metabolism in cancer	2.88E-02	4.37E-02	RAC2;SLC22A3
**cluster8**			
Antigen Presentation: Folding, assembly and peptide loading of class I MHC	1.86E-03	5.27E-03	HLA C;HLA G
Autoimmune thyroid disease	8.83E-03	1.77E-02	HLA C;HLA G
Allograft rejection	4.62E-03	1.16E-02	HLA C;HLA G
Graft versus host disease	5.36E-03	1.18E-02	HLA C;HLA G
Type I diabetes mellitus	5.88E-03	1.26E-02	HLA C;HLA G
Proteasome Degradation	1.27E-02	2.37E-02	HLA C;HLA G
Cellular senescence	8.86E-03	1.73E-02	HLA C;HLA G;TRPV4
Viral carcinogenesis	1.64E-02	2.88E-02	HLA C;HLA G;SP100
Human T cell leukemia virus 1 infection	2.05E-02	3.54E-02	HLA C;HLA G;IL15
Immunoregulatory interactions between a Lymphoid and a non Lymphoid cell	2.10E-02	3.56E-02	HLA C;HLA G;IFITM1
Allograft Rejection	2.46E-02	3.87E-02	HLA C;HLA G
**cluster9**			
JAK STAT MolecularVariation 1	2.25E-03	6.01E-03	IL12RB2;IL15;TNFSF10
Cytokine cytokine receptor interaction	4.35E-02	5.98E-02	IL12RB2;IL15;TNFSF10
JAK STAT pathway and regulation	4.96E-02	6.61E-02	IL12RB2;IL15;TNFSF10
**cluster10**			
Rheumatoid arthritis	2.41E-02	3.86E-02	CTSL;IL15
TNF signaling pathway	3.49E-02	5.04E-02	IL15;MLKL

**Figure 6:**
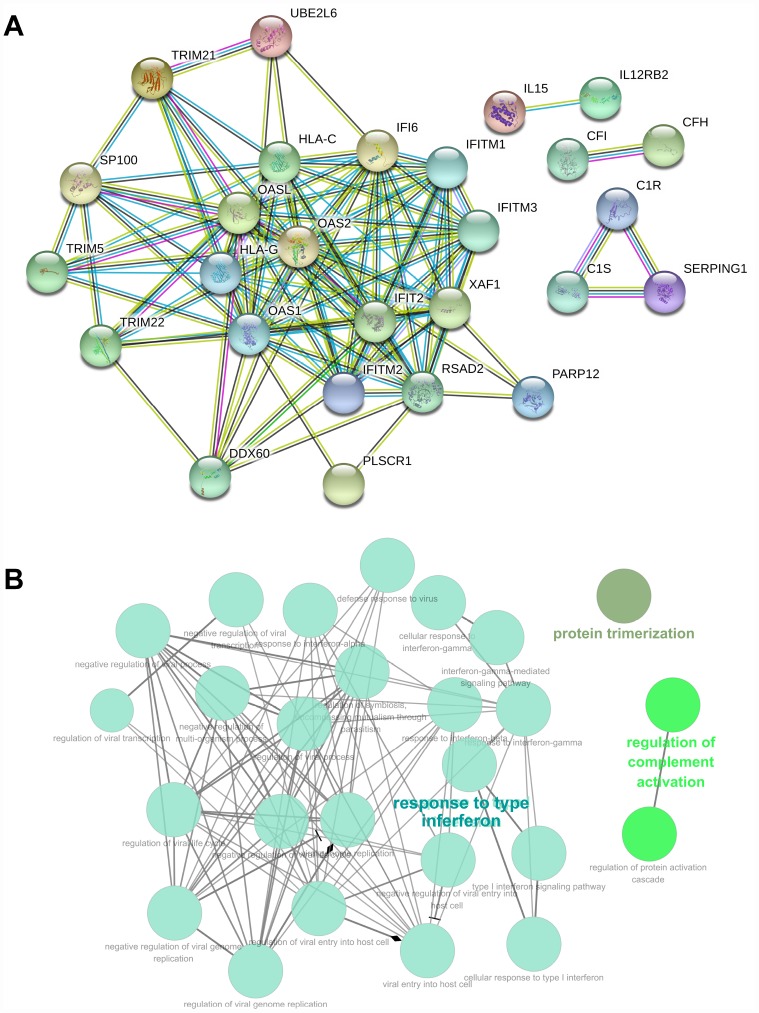
**PPI and GO analysis involved in the co-expression Pink module.** (**A**) PPI analysis involved in the co-expression Pink module. (**B**) GO analysis involved in the co-expression Pink module.

The 73 genes in the co-expression Pink module were uploaded to STRING for protein–protein interaction (PPI) analysis (Figure 6A). The combined scores of nodes ranged from 0.400 to 0.999. The PPI results also determined co-expression coefficient between proteins (Supplementary Table 7). High combined scores and co-expression coefficients indicated that there were interaction effects on spatial position and expression events between proteins. Some of interaction results showed both high combined scores (value > 0.9) and high co-expression coefficients (value > 0.8), such as RSAD2 and OASL, RSAD2 and XAF1, DDX60 and RSAD2, RSAD2 and IFIT2, IFITM2 and IFITM1, OAS1 and IFI6, C1R and C1S, IFITM2 and IFITM3 (Supplementary Table 7). Some interaction results showed high combined scores (value > 0.9), but low co- expression coefficients (value = 0), including TRIM5 and HLA-C, HLA-G and IFI6, HLA-G and IFITM3, IFITM2 and HLA-G, HLA-G and IFITM1, HLA-G and XAF1, HLA-G and OAS2, HLA-G and IFIT2, HLA-G and TRIM5, HLA-G and TRIM21, HLA-G and TRIM22, HLA-C and XAF1, TRIM22 and HLA-C. This indicated that interaction effects between proteins were not only at the level of expression, but also signaling cascade.

GO enrichment analysis of genes also revealed biological processes in the co-expression Pink module (Supplementary Table 8). The genes in the co-expression Pink module were mainly distributed in three parts, including protein trimerization, regulation of complement activation, and response to interferon. Among the biological processes, the responses to interferon also included many detailed functions. This includes: negative regulation of multi-organism process, regulation of symbiosis, encompassing mutualism through parasitism, response to type-I interferon, response to interferon-gamma, negative regulation of viral process, regulation of viral process, defense response to virus, response to interferon-alpha, response to interferon-beta, viral genome replication, cellular response to interferon-gamma, cellular response to type I interferon, regulation of viral life cycle, negative regulation of viral life cycle, negative regulation of viral transcription, regulation of viral transcription, interferon-gamma-mediated signaling pathway, type I interferon signaling pathway, regulation of viral genome replication, negative regulation of viral genome replication, viral entry into host cell, regulation of viral entry into host cell, and negative regulation of viral entry into host cell (Figure 6B). We found consistency was between KEGG and GO analysis, which indicated the function of tumor immunity.

### Network analysis and survival-associated lncRNAs

**Figure 7:**
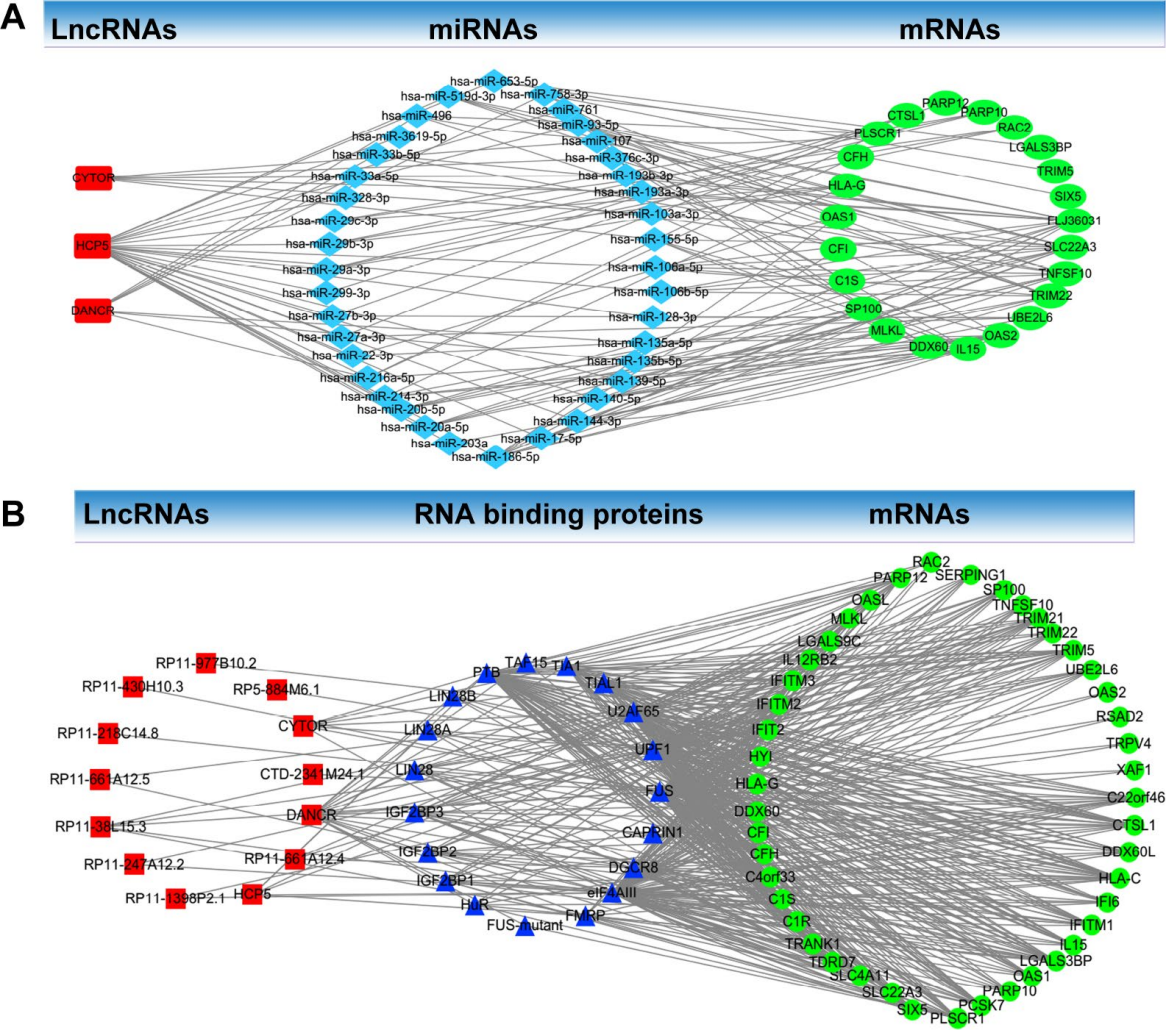
**LncRNA-RNA binding protein-mRNA network and LncRNA- miRNA-mRNA network.** (**A**) LncRNA-RNA binding protein-mRNA network based on the co-expression Pink module. (**B**) LncRNA-miRNA-mRNA network based on the co-expression Pink module.

A ceRNA network analysis was used to determine whether lncRNAs regulate the identified mRNAs in the co-expression Pink module through miRNAs. The ceRNA network analysis found three lncRNAs (CYTOR, HCP5, and DANCR) in the co-expression Pink module: 33 miRNAs (hsa-miR-106a-5p, hsa-miR-106b-5p, hsa-miR-128-3p, hsa-miR-135a-5p, hsa-miR-135b-5p, hsa-miR-139-5p, hsa-miR-140-5p, hsa-miR-144-3p, hsa-miR-17-5p, hsa-miR-186-5p, hsa-miR-203a, hsa-miR-20a-5p, hsa-miR-20b-5p, hsa-miR-214-3p, hsa-miR-216a-5p, hsa-miR-216a-5p, hsa-miR-22-3p, hsa-miR-27a-3p, hsa-miR-27b-3p, hsa-miR-299-3p, hsa-miR-29a-3p, hsa-miR-29b-3p, hsa-miR-29c-3p, hsa-miR-328-3p, hsa-miR-33a-5p, hsa-miR-33b-5p, hsa-miR-3619-5p, hsa-miR-496, hsa-miR-519d-3p, hsa-miR-653-5p, hsa-miR-758-3p, hsa-miR-761, hsa-miR-93-5p). 23 identified mRNAs (FLJ36031, SLC22A3, TNFSF10, TRIM22, UBE2L6, OAS2, IL15, DDX60, MLKL, SP100, C1S, CFI, OAS1, HLA-G, CFH, PLSCR1, CTSL1, PARP12, PARP10, RAC2, LGALS3BP, TRIM5, SIX5) in the co-expression Pink module were involved in a ceRNA network (Figure 7A).

LncRNA-RNA binding protein-mRNA network analysis was used to determine whether lncRNAs regulate identified mRNAs through RNA binding proteins. Through this analysis, we found in the co-expression Pink module 13 lncRNAs (CYTOR, CTD-2341M24.1, DANCR, RP11-661A12.4, HCP5, RP11-1398P2.1, RP11-247A12.2, RP11-38L15.3, RP11-661A12.5, RP11-218C14.8, RP11-430H10.3, RP11-977B10.2, RP5-884M6.1). We also found 19 RNA-binding proteins (FUS, CAPRIN1, DGCR8, eIF4AIII, FMRP, FUS-mutant, HuR, IGF2BP1, IGF2BP2, IGF2BP3, LIN28, LIN28A, LIN28B, PTB, TAF15, TIA1, TIAL1, U2AF65, UPF1) and 45 identified mRNAs (C22orf46, CTSL1, DDX60L, HLA-C, IFI6, IFITM1, IL15, LGALS3BP, OAS1, PARP10, PCSK7, PLSCR1, SIX5, SLC22A3, SLC4A11, TDRD7, TRANK1, C1R, C1S, C4orf33, CFH, CFI, DDX60, HLA-G, HYI, IFIT2, IFITM2, FITM3, IL12RB2, LGALS9C, MLKL, OASL, PARP12, RAC2, SERPING1, SP100, TNFSF10, TRIM21, TRIM22, TRIM5, UBE2L6, OAS2, RSAD2, TRPV4, XAF1) in the co-expression Pink module which were involved in the network (Figure 7B).

**Figure 8:**
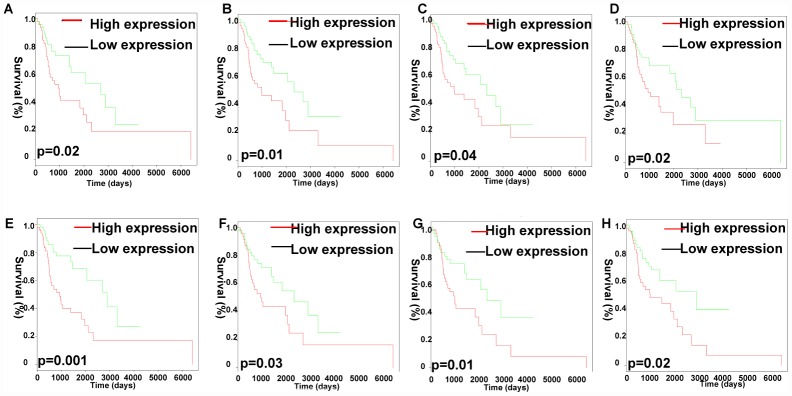
**Analysis of OS-related identified lncRNAs in the co-expression Pink module.** Kaplan-Meier survival analysis of RP11-977B10.2 (**A**), RP11-661A12.5 (**B**), RP11-661A12.4 (**C**), RP11-646E18.4 (**D**), RP11-247A12.2 (**E**), RP1-137D17.2 (**F**), MIR4435-2HG (**G**), CYTOR (**H**). X-axis represented survival time (days), and y-axis represented survival rate.

Furthermore, the Kaplan Meier-plotter analysis revealed that 8 out of the 26 identified lncRNAs in the co-expression Pink module were significantly associated with overall survival (p < 0.05), including CYTOR (p=0.0202), MIR4435-2HG (p=0.0169), RP1-137D17.2 (p=0.0340), RP11-247A12.2 (p=0.0016), RP11-646E18.4 (p=0.0286), RP11-661A12.4 (p=0.0417), RP11-661A12.5 (p=0.0134), RP11-977B10.2 (p=0.0201) (Figure 8).

### RT-qPCR validation of identified molecules

**Figure 9:**
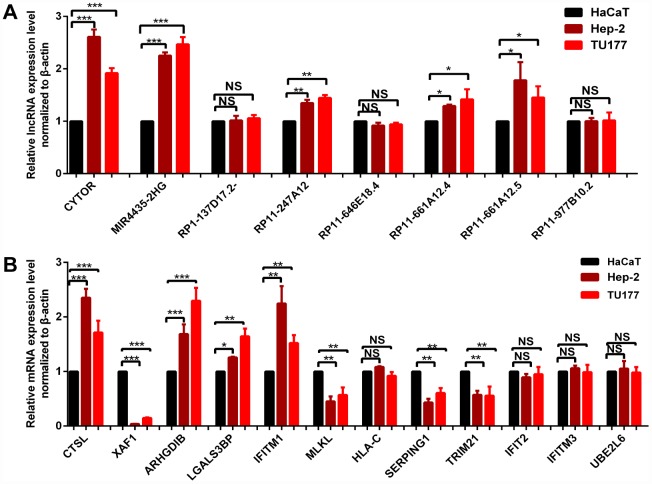
qRT-PCR validation of 8 survival-related lncRNAs (**A**) and 12 hub-mRNAs (**B**) in LC cell models compared to control cells. * p < 0.05. ** p < 0.01. *** p < 0.001.

qRT-PCR was used to validate the expressions of survival-associated lncRNAs and hub mRNAs resulting from WGCNA analysis, including 8 lncRNAs (CYTOR, MIR4435-2HG, RP1-137D17.2, RP11-247A12.2, RP11-646E18.4, RP11-661A12.4, RP11-661A12.5, and RP11-977B10.2) and 12 hub mRNAs (IFIT2, XAF1, UBE2L6, IFITM3, HLA-C, CTSL, ARHGDIB, LGALS3BP, IFITM1, MLKL, SERPING1, TRIM21) in cultured LC cells (Hep-2 and TU177) and one control cell (HaCaT keratinocytes) (Figure 9) [23]. The results indicated significant difference for five survival-associated lncRNAs (CYTOR, MIR4435-2HG, RP11-247A12.2, RP11-661A12.4, and RP11-661A12.5) (Figure 9A), and eight hub mRNAs (CTSL, XAF1, ARHGDIB, LGALS3BP, IFITM1, MLKL, SERPING1, and TRIM21) (Figure 9B) between LC cells and control cells.

## DISCUSSION

Even with advancement in medical technology, the five year survival rate was still devastating in advanced LC patients. Various factors can increase the risk of LC; smoking is a leading cause [24]. There is also evidence of a link between consumption of alcohol without food and high incidence of LC [25]. Additionally, postoperative N stage was one of independent prognostic factors for patients with LC after curative resection [26]. Postoperative radiotherapy was also studied as a prognostic factor, and it is indicated that accelerated hyperfraction radiotherapy is better than conventional fractionation radiotherapy for early glottic cancer based on the optimal data [27]. Furthermore, new targeted therapy of laryngeal cancer provides fresh insight into treatment for LC patients. LncRNA plays a key role in various biological processes including transcriptional regulation, chromatin modification, RNA editing, posttranscriptional processing, and molecular metabolism. In the previous study, the identified lncRNA ST7-AS1 and lncRNA UCA1in LC cells promoted tumor progression [28, 29]. Regarding mechanism research, lncRNA-miRNA- mRNA axis or lncRNA-RNA binding protein-mRNA axis was also studied in LC. LncRNA SNHG1 is significantly upregulated in LC and associated with prognosis of LC patients through activation of the miR-375/YAP1/Hippo signaling axis [30]. LncRNA NEAT1 promotes laryngeal squamous cell cancer through regulation of the miR-107/CDK6 pathway [31].

In this study, a total of 9 co-expression gene modules were constructed by 5000 good genes (including 2500 lncRNAs and 2500 mRNAs) from 99 human LC samples. The samples were provided by WGCNA, which was used to identify significant gene modules in relation to important clinical phenotypes. WGCNA was performed to screen the clusters of co-expressed genes to identify prognostic biomarkers in breast cancer and also to obtain hub genes. In one prognostic study performed by WGCNA, a significant negative correlation between the high expression of PYCR1 and TRPM2-AS and the breast cancer survival was elucidated [32]. In other groups, WGCNA and PPI network analysis were applied to identify hub genes correlated with bladder cancer progression to explore the underlying disease mechanisms, and identify more effective biomarkers for bladder cancer. Survival analyses of the identified genes indicated that elevated expressions of six potential biomarkers, including COL3A1, FN1, COL5A1, FBN1, COL6A1 and THBS2 were significantly associated with a worse OS [33]. So far, there has only been one study applying WGCNA to study the mechanisms of LC. The microarray of GSE51958, including 10 samples, was analyzed in this study by WGCNA package in R. The results showed that TPX2, MCM2, UHRF1, CDK2 and PRC1 were found to have a possible association with LC [34]. In our study, the co-expression Pink module was significantly correlated with four clinical traits, including history of smoking, lymph node count, tumor status, and success of follow-up treatment.

In our analysis, we found some consistency with previous reports. For example, the identified immune cytokine tumor necrosis factor-related apoptosis-inducing ligand (TRAIL) in the co-expression Pink module has received high attention as a promising drug due to its ability to trigger cancer cell apoptosis and anti-tumor immune response without causing toxicity *in vivo* [35]. IL-15 is an inflammatory cytokine that plays an essential role in the development and activation of natural killer (NK) cells [36]. In a recent study, the activation of the IL-15 signaling in adipose tissue probably activated and expanded the NK cell population and inhibited tumor growth [37]. HLA-G expression was increased in benign and premalignant lesions, and gradually decreased in invasive carcinomas and in respective draining cervical lymph nodes. The expression of the nonclassical HLA-G molecules in laryngeal lesions was reported as biomarkers of tumor invasiveness [38]. However, we also made some new discoveries that were not previously reported in LC, such as CFH, C1R, CFI, C1S, SP100, OAS1, IL15, OAS2, RAC2, RSAD2, TRIM21. The newly identified biomarkers in LC showed important functions. For example, TRIM21 mediates ubiquitination of Snail and modulates epithelial to mesenchymal transition in breast cancer cells [39]. RAC2 promotes abnormal proliferation of quiescent cells by enhanced JUNB expression via the MAL-SRF pathway.

Furthermore, the ceRNA network and identification of integrated lncRNA-RNA binding protein-mRNA signatures were constructed for mechanism research in LC. Some of lncRNAs were reported in previous studies and closely related to the proliferation, apoptosis, angiogenesis, invasiveness, and migration of cancer [40]. For example, lncRNA DANCR was significantly upregulated in bladder cancer tissues and cases with lymph node metastasis, late tumor stage, high histological grade, and poor patient prognosis [41]. LncRNA HCP5 is frequently downregulated in human ovarian cancer, suggesting that HCP5 may be involved in the pathogenesis of the disease [42]. Additionally, the K-M plot analysis revealed that 8 out of the identified lncRNAs in the co-expression Pink module were significantly associated with OS of LC patients, including CYTOR, MIR4435-2HG, RP1-137D17.2, RP11-247A12.2, RP11-646E18.4, RP11-661A12.4, RP11-661A12.5, RP11-977B10.2. MIR4435-2HG promoted cell proliferation and tumorigenesis in gastric cancer, non-small cell lung cancer and breast cancer [43–45]. CYTOR modulated proliferation, migration, invasion of colorectal cancer, head and neck squamous cell carcinoma [46, 47]. The other identified lncRNAs were not reported in previous studies, and therefore our work adds valuable data indentifying new lncRNA biomarkers to the field of LC.

We do acknowledge the limitations of our study. In this study, there were LC patients with incomplete clinical information, which might affect the clinical assessment of the research result. Second, the identified lncRNAs and mRNAs were confirmed in cell culture models; therefore, it might be also necessary to further validate these results in large-scale clinical samples for their real clinical application. Finally, although the sample size (n=99) was acceptable for analysis of hub molecules and survival analysis in the mRNA level, it does not account for the final expression of these proteins. It would be necessary to further verify those hub molecule biomarkers in the protein level in clinical samples.

## CONCLUSIONS

In this study, we used WGCNA to determine that the co-expression Pink module was significantly correlated with four clinical traits. These obtained genes (including lncRNAs and mRNAs) from the co-expression modules enriched in various pathways and cell functions were closely related with the risk factors and development progress of LC. Furthermore, we elucidated a series of new biomarkers which could be useful for the diagnosis and treatment of LC.

## MATERIALS AND METHODS

### TCGA data of LC patients

Data of LC patients was obtained from the TCGA database (http://cancergenome.nih.gov/). Level 3 RNA-seq V2 (including lncRNA and mRNA expression data) and clinical data of 99 LC patients was downloaded. The basic pretreatment method of RNA-seq data is to remove genes where the missing value (expression = 0) is more than 20%. OS analysis was performed with R survival package, according to genes expression data (cutoff value = median value of gene expression) and survival time in LC patients. 11 LC clinical traits were extracted, including age at initial pathologic diagnosis (Patients were aged 38 to 83), history of smoking (from 0.9 packs to 150 packs), history of alcohol consumption (yes or no), intermediate dimension (from 0.2cm-1.4cm), lymph node count (from 0 to 104), neck lymph node dissection (yes or no), pathologic N stage (N0, N1, N2, N3, and NX), radiation therapy (yes or no), targeted molecular therapy (yes or no), tumor status (with tumor or tumor free), and success of follow-up treatment (complete remission/response, partial remission/ response, persistent disease, progressive disease, and stable disease).

### Weighted correlation network analysis of lncRNAs and mRNAs

Based on the processed TCGA data in LC patients, the top 2500 lncRNAs and 2500 mRNA were selected as good genes for further WGCNA analysis. Those 5000 genes were normalized by Limma package with Voom function, then the auxiliary data was removed and expression data was transposed for further analysis. First, all samples were checked for outliers with flashClust to construct sampleTree. The outlier in the sample was then removed based on cutHeight. A sample dendrogram and trait heatmap were visualized by WGCNA package of R software (http://www.r-project.org/) to develop networks to investigate the relationship between the corresponding sample gene expression data and clinical phenotypes [15]. The adjacency matrix aij that calculated the connection strength between each pair of nodes was measured as follows: sij = |cor(xi, xj)| aij = Sijβ. Xi and Xj were vectors of expression values for genes *i* and *j*, sij represented the Pearson’s correlation coefficient of genes I and j, aij encoded the network connection strength between genes i and j. β value was the soft-threshold (power value). Moreover, the Scale-Free Topology Fit Index (SFTFI) (scale free R^2^) ranging from 0 to 1 was used to determine a scale-free topology model. Choosing a set of soft-thresholding powers (range: 1 to 20) can help calculate the scale free topology model fit [16]. In this study, we defined the adjacency matrix using soft thresholding with beta=5, and the corresponding scale free R2 value was 0.87 to obtain a good scale-free topology model. In the co-expression network, genes with highly absolute correlations were clustered into the same module to generate a cluster dendrogram (The parameters are described below: TOMType = unsigned method, minModuleSize = 30, reassignThreshold = 0, mergeCutHeight = 0.25). The dendrogram can be displayed together with the color assignment to form the network heatmap plot using the adjacency matrix algorithm method. The average linkage hierarchical clustering was conducted according to Topological Overlap Matrix (TOM)-based dissimilarity measure. Heatmap tool package was plotted to analyze the strength of network interactions. The relationships between co-expression modules and 11 LC clinical traits were calculated by the Pearson correlation coefficient and plotted by heat map. In addition, the Gene Significance (GS) was defined as mediated *p*-value of each gene (GS = lgP) in the linear regression between gene expression and the clinical traits.

### Bioinformatics analysis provided insight into pathways and potential functions

The pathway (http://ci.smu.edu.cn/genclip3/analysis.php) and Gene Ontology (GO) (http://www.cytoscape.org/) enrichment analyses within the co-expression modules were performed to identify LC-related pathway and biological functions, with an adjusted *p* value < 0.05 (Benjamini-Hochberg for multiple testing). Furthermore, the LC survival-related lncRNAs in the co-expression Pink module were plotted with Rstudio.

### Identification of hub molecules with molecular complex detection

The mRNA-mRNA interactions were constructed by STRING and analyzed with Cytoscape software (version 3.2.0; National Resource for Network Biology) to obtain the hub genes. The hub genes were extracted from the identified clinical-related co-expression modules using Molecular Complex Detection. MCODE plugin is a reliable method to distinguish hub molecules from non-hub molecules. The criteria for hub-molecule searching was set as the molecular complex detection (MCODE) score > 6, and statistical significance of *p* < 0.05 [17].

### The ceRNA network and identification of integrated lncRNA-RNA binding protein-mRNA signatures

StarBase provides systematical data of the RNA-RNA and protein-RNA interaction networks from 108 CLIP-Seq (PAR-CLIP, HITS-CLIP, iCLIP, CLASH) data sets generated by 37 independent studies [48]. LncRNA-miRNA-mRNA and lncRNA-RNA binding protein-mRNA interaction networks based on the co-expression modules were decoded from the large-scale CLIP-Seq data by starBase v2.0 (http://starbase.sysu.edu.cn/starbase2/index.php). Network visualization was performed with Cytoscape 3.4.0 (http://www.cytoscape.org/).

### Cell lines and cell culture

LC cell lines Hep-2, TU177 cells and normal control cell line HaCaT keratinocytes cells were purchased from Keibai Academy of Science (Nanjing, China). Hep-2 cells were cultured in EMEM medium, TU177 cells were cultured in 1640 medium, and HaCaT cells were in DMEM medium (Corning, NY, USA) supplemented with 10% fetal bovine serum (FBS, Gibco). All these cells were maintained with 5% CO2 atmosphere at 37°C [23].

### RNA extraction and qRT-PCR

Total RNAs were extracted from cell lines with TRizol Reagent (Invitrogen) according to the manufacturer’s instructions. Total RNAs were reversely transcribed into cDNAs and then used to perform quantitative real-time PCR (qRT-PCR) with SYBR Premix ExTaq (TaKaRa). Beta-actin was used as an internal control for gene quantification. The RNA molecules that were assessed on the cell lines and their corresponding primers were listed in Supplementary Table 9.

### Statistical analysis

All data was analyzed by R software 3.4.3 (https://www.r-project.org/). In all cases, *p* < 0.05 was considered statistically significance. For KEGG pathway and GO analysis, Pearson correlation coefficient was calculated, and Benjamin-Hochberg was used for multiple testing and calculated to adjust the *p*-value.

## Supplementary Material

Supplementary Tables 4, 5, 6, 8, 9

Supplementary Table 1

Supplementary Table 2

Supplementary Table 3

Supplementary Table 7
