# Supplementary material for: Characterization of long non-coding RNA and messenger RNA profiles in laryngeal cancer by weighted gene co-expression network analysis
Source: Aging (Albany NY). 2019 Nov 18;11(22):10074–99. doi: 10.18632/aging.102419 (PMC6914418; doi:10.18632/aging.102419)
Supplement: Supplementary Tables 4, 5, 6, 8, 9 [file aging-11-102419-s003..pdf]

SUPPLEMENTARY TABLES

Supplementary Table 4. Analysis of network topology for various softthresholding powers.

Power	SFT.R.^{sq}	slope	truncated.R.^{sq}	mean.k.	median.k.	max.k.
1	0.7	11.3	0.784	723	7.07E+02	1190
2	0.156	0.669	0.979	170	1.55E+02	440
3	0.65	-1.56	0.986	52.6	4.29E+01	206
4	0.811	-1.91	0.969	19.7	1.37E+01	112
5	0.87	-1.95	0.975	8.56	5.04E+00	67.5
6	0.898	-1.93	0.986	4.18	2.06E+00	43.8
7	0.91	-1.9	0.986	2.25	1.08E+00	30.1
8	0.91	-1.84	0.975	1.32	5.59E-01	21.7
9	0.882	-1.65	0.959	0.839	2.87E-01	16.2
10	0.814	-1.52	0.897	0.57	1.55E-01	12.4
12	0.91	-1.8	0.932	0.316	4.75E-02	7.67
13	0.86	-1.67	0.888	0.254	2.80E-02	6.19
14	0.82	-1.48	0.866	0.214	1.65E-02	5.06
15	0.759	-1.24	0.854	0.186	9.72E-03	4.36
16	0.792	-1.62	0.812	0.167	5.78E-03	3.82
17	0.801	-1.45	0.881	0.153	3.51E-03	3.37
18	0.772	-1.32	0.848	0.143	2.19E-03	2.99
19	0.737	-1.15	0.816	0.135	1.39E-03	2.66
20	0.699	-0.824	0.826	0.129	8.55E-04	2.38

Note:SFT= scale-free topology;sq=square,k=number of clusters.

Supplementary Table 5. List of genes in the co-expression pink module.

Gene ID	Description
AP001610.5	long intergenic non-protein coding RNA,AP001610.5
C1R	complement C1r
C1S	complement C1s
C22orf46	chromosome 22 open reading frame 46
C4orf33	chromosome 4 open reading frame 33
CFH	complement factor H
CFI	complement factor I
CTD-2341M24.1	long non-coding RNA CTD-2341M24.1
CTSL1	cathepsin L
CYTOR	cytoskeleton regulator RNA
DANCR	differentiation antagonizing non-protein coding RNA
DDX60	DExD/H-box helicase 60
DDX60L	DExD/H-box 60 like
FLJ36031	coiled-coil domain containing 71 like, C7orf74
HCP5	HLA complex P5
HLA-C	major histocompatibility complex, class I, C
HLA-G	major histocompatibility complex, class I, G
HYI	hydroxypyruvate isomerase (putative)
IFI6	interferon alpha inducible protein 6
IFIT2	interferon induced protein with tetratricopeptide repeats 2
IFITM1	interferon induced transmembrane protein 1
IFITM2	interferon induced transmembrane protein 2
IFITM3	interferon induced transmembrane protein 3
IL12RB2	interleukin 12 receptor subunit beta 2
IL15	interleukin 15
LGALS3BP	galectin 3 binding protein
LGALS9C	galectin 9C
LINC01869	long intergenic non-protein coding RNA 1869
LINC01932	long intergenic non-protein coding RNA 1932
LINC02012	long intergenic non-protein coding RNA 2012
LINC02100	long intergenic non-protein coding RNA 2100
MIR4435-2HG	MIR4435-2 host gene
MLKL	mixed lineage kinase domain like pseudokinase
OAS1	2'-5'-oligoadenylate synthetase 1
OAS2	2'-5'-oligoadenylate synthetase 2
OASL	2'-5'-oligoadenylate synthetase like
PARP10	poly(ADP-ribose) polymerase family member 10
PARP12	poly(ADP-ribose) polymerase family member 12
PCSK7	proprotein convertase subtilisin/kexin type 7
PLSCR1	phospholipid scramblase 1
RAC2	Rac family small GTPase 2
RP11-1398P2.1	long non-coding RNA RP11-1398P2.1
RP11-20D14.6	long non-coding RNA RP11-20D14.6

RP11-218C14.8	long non-coding RNA RP11-218C14.8
RP11-247A12.2	long non-coding RNA RP11-247A12.2
RP11-288L9.4	long non-coding RNA RP11-288L9.4
RP11-326G21.1	long non-coding RNA RP11-326G21.1
RP1-137D17.2	long non-coding RNA RP1-137D17.2
RP11-38L15.3	long non-coding RNA RP11-38L15.3
RP11-430H10.3	long non-coding RNA RP11-430H10.3
RP11-646E18.4	long non-coding RNA RP11-646E18.4
RP11-661A12.4	long non-coding RNA RP11-661A12.4
RP11-661A12.5	long non-coding RNA RP11-661A12.5
RP11-977B10.2	long non-coding RNA RP11-977B10.2
RP5-1185I7.1	long non-coding RNA RP5-1185I7.1
RP5-884M6.1	long non-coding RNA RP5-884M6.1
RSAD2	radical S-adenosyl methionine domain containing 2
SERPING1	serpin family G member 1
SIX5	SIX homeobox 5
SLC22A3	solute carrier family 22 member 3
SLC4A11	solute carrier family 4 member 11
SP100	SP100 nuclear antigen
TDRD7	tudor domain containing 7
TNFSF10	TNF superfamily member 10
TRANK1	tetratricopeptide repeat and ankyrin repeat containing 1
TRIM21	tripartite motif containing 21
TRIM22	tripartite motif containing 22
TRIM5	tripartite motif containing 5
TRPV4	transient receptor potential cation channel subfamily V member 4
UBE2L6	ubiquitin conjugating enzyme E2 L6
XAF1	XIAP associated factor 1
ZNF649-AS1	ZNF649 antisense RNA 1, non coding RNA
ZNRD1ASP	zinc ribbon domain containing 1 antisense, non coding RNA

Supplementary Table 6. The hub genes in the co-expression pink module.

Hub genes	Description
IFIT2	interferon induced protein with tetratricopeptide repeats 2
XAF1	XIAP associated factor 1
UBE2L6	ubiquitin conjugating enzyme E2 L6
IFITM3	interferon induced transmembrane protein 3
HLA-C	major histocompatibility complex, class I, C
CTSL	cathepsin L
ARHGDIB	Rho GDP dissociation inhibitor beta
LGALS3BP	galectin 3 binding protein
IFITM1	interferon induced transmembrane protein 1
MLKL	mixed lineage kinase domain like pseudokinase
SERPING1	serpin family G member 1
TRIM21	tripartite motif containing 21

Supplementary Table 8. Gene ontology analysis that are involved with the genes in the co-expression pink module.

GO ID	GO term	Group P value	Group PValue corrected with benjamini-hochberg	% Associated genes	Nr. genes	Associated genes found
GO:0070206	protein trimerization	0.00	0.00	6.78	4.00	[MLKL, TRIM21, TRIM22, TRIM5]
GO:2000257	regulation of protein activation cascade	0.00	0.00	4.07	5.00	[C1R, C1S, CFH, CFI, SERPING1]
GO:0030449	regulation of complement activation	0.00	0.00	4.13	5.00	[C1R, C1S, CFH, CFI, SERPING1]
GO:0043901	negative regulation of multi-organism process	0.00	0.00	6.32	11.00	[IFITM1, IFITM2, IFITM3, OAS1, OASL, PARP10, PLSCR1, RSAD2, SP100, TRIM21, TRIM5]
GO:0043903	regulation of symbiosis, encompassing mutualism through parasitism	0.00	0.00	4.64	11.00	[IFITM1, IFITM2, IFITM3, OAS1, OASL, PARP10, PLSCR1, RSAD2, SP100, TRIM21, TRIM5]
GO:0034340	response to type I interferon	0.00	0.00	14.29	13.00	[HLA-C, HLA-G, IFI6, IFIT2, IFITM1, IFITM2, IFITM3, OAS1, OAS2, OASL, RSAD2, SP100, XAF1]
GO:0034341	response to interferon-gamma	0.00	0.00	6.78	12.00	[HLA-C, HLA-G, IFITM1, IFITM2, IFITM3, OAS1, OAS2, OASL, SP100, TRIM21, TRIM22, TRIM5]

GO:0048525	negative regulation of viral process	0.00	0.00	11.96	11.00	[IFITM1, IFITM2, IFITM3, OAS1, OASL, PARP10, PLSCR1, RSAD2, SP100, TRIM21, TRIM5]
GO:0050792	regulation of viral process	0.00	0.00	5.42	11.00	[IFITM1, IFITM2, IFITM3, OAS1, OASL, PARP10, PLSCR1, RSAD2, SP100, TRIM21, TRIM5]
GO:0051607	defense response to virus	0.00	0.00	5.56	13.00	[DDX60, IFIT2, IFITM1, IFITM2, IFITM3, IL15, OAS1, OAS2, OASL, PLSCR1, RSAD2, TRIM22, TRIM5]
GO:0035455	response to interferon-alpha	0.00	0.00	20.83	5.00	[IFIT2, IFITM1, IFITM2, IFITM3, OAS1]
GO:0035456	response to interferon-beta	0.00	0.00	21.74	5.00	[IFITM1, IFITM2, IFITM3, PLSCR1, XAF1]
GO:0019079	viral genome replication	0.00	0.00	7.21	8.00	[IFITM1, IFITM2, IFITM3, OAS1, OASL, PARP10, PLSCR1, RSAD2]
GO:0071346	cellular response to interferon-gamma	0.00	0.00	5.73	9.00	[HLA-C, HLA-G, OAS1, OAS2, OASL, SP100, TRIM21, TRIM22, TRIM5]
GO:0071357	cellular response to type I interferon	0.00	0.00	14.94	13.00	[HLA-C, HLA-G, IFI6, IFIT2, IFITM1, IFITM2, IFITM3, OAS1, OAS2, OASL, RSAD2, SP100, XAF1]
GO:1903900	regulation of viral life cycle	0.00	0.00	7.19	10.00	[IFITM1, IFITM2, IFITM3, OAS1, OASL, PARP10, PLSCR1, RSAD2, TRIM21, TRIM5]
GO:1903901	negative regulation of viral life cycle	0.00	0.00	13.33	10.00	[IFITM1, IFITM2, IFITM3, OAS1, OASL, PARP10, PLSCR1, RSAD2, TRIM21, TRIM5]
GO:0032897	negative regulation of viral transcription	0.00	0.00	12.00	3.00	[IFITM3, SP100, TRIM21]
GO:0046782	regulation of viral transcription	0.00	0.00	4.41	3.00	[IFITM3, SP100, TRIM21]
GO:0060333	interferon-gamma-mediated signaling pathway	0.00	0.00	9.28	9.00	[HLA-C, HLA-G, OAS1, OAS2, OASL, SP100, TRIM21, TRIM22, TRIM5]
GO:0060337	type I interferon signaling pathway	0.00	0.00	14.94	13.00	[HLA-C, HLA-G, IFI6, IFIT2, IFITM1, IFITM2, IFITM3, OAS1, OAS2, OASL, RSAD2, SP100,

						XAF1]
GO:0045069	regulation of viral genome replication	0.00	0.00	9.64	8.00	[IFITM1, IFITM2, IFITM3, OAS1, OASL, PARP10, PLSCR1, RSAD2]
GO:0045071	negative regulation of viral genome replication	0.00	0.00	15.69	8.00	[IFITM1, IFITM2, IFITM3, OAS1, OASL, PARP10, PLSCR1, RSAD2]
GO:0046718	viral entry into host cell	0.00	0.00	4.07	5.00	[IFITM1, IFITM2, IFITM3, TRIM21, TRIM5]
GO:0046596	regulation of viral entry into host cell	0.00	0.00	14.71	5.00	[IFITM1, IFITM2, IFITM3, TRIM21, TRIM5]
GO:0046597	negative regulation of viral entry into host cell	0.00	0.00	20.00	4.00	[IFITM1, IFITM2, IFITM3, TRIM5]

Supplementary Table 9. The list of RNA molecules that were assessed on the cell lines.

RNA type	Primer name	Primer sequence (from 5′ to 3′)
lncRNA	CYTOR-F	CCACATTCCAACCTCCGTCTGC
	CYTOR-R	TCGGCGGGCAACAGGTAGAG
	MIR4435-2HG-F	GTTGCCCGCCGATCACAGC
	MIR4435-2HG-R	ACCGACCAGACCAGCCCATG
	RP1-137D17.2-F	CCAAGTTCTGCTGCTGCCTCAG
	RP1-137D17.2-R	TCCCTCCCATGCCCTGAAACC
	RP11-247A12.2-F	AGGCTGGAGTGCGGTGGTG
	RP11-247A12.2-R	GGGAGGGTGAGGCAGGAGAATC
	RP11-646E18.4-F	GGGAGGGCTAGTCACGGAACG
	RP11-646E18.4-R	CCTCAGCCTCTGCTCCTCCAC
	RP11-661A12.4-F	ACCAGAAGGGGCTACCGCATAG
	RP11-661A12.4-R	TTGGCGAGAGAGCAGAGGTCAG
	RP11-661A12.5-F	AGAGCAGGAGAAGACGCAGGAG
	RP11-661A12.5-R	TGATCTGTGCAGTGCGGTTGTC
	RP11-977B10.2-F	GGTCTTGAGTGGGGCAATCAGC
	RP11-977B10.2-R	GAGGTCTTTGCAGGAGCCGATG
mRNA	IFIT2-F	AACCTACTGGCCTATCTAAAGC
	IFIT2-R	CATGCTCTTGCTGGATTAACTC
	XAF1-F	AAGAGGTTCTGGTTTCAGGAAA
	XAF1-R	CTGAGCTTGAGTATCTCCAGAG
	UBE2L6-F	AGCTGGAGGATCTTCAGAAGAA
	UBE2L6-R	TGGTTGTGAATTTGATCATGGG
	IFITM3-F	CTTCTTCTCTCCTGTCAACAGT
	IFITM3-R	GTTCATGAAGAGGGTGTTGAAC
	HLA-C-F	CTACGACGGCAAGGATTACATC
	HLA-C-R	CTCATGGTCAGAGACGAGATG
	CTSL-F	TATTTTGAGCCAGACTGTAGCA
	CTSL-R	GATTCTGTGCTTTCAAATCCGT
	ARHGDIB-F	GCAAGCTCAATTATAAGCCTCC
	ARHGDIB-R	CTTCCAGATCTCCAGTAAGGTC
	LGALS3BP-F	CAATGGTACTTCTACTCCCGAA
	LGALS3BP-R	GAACTGTAGGCAGAGCTTCTC
	IFITM1-F	GTTCAACACCCTCTTCTTGAAC
	IFITM1-R	CATCTTCCTGTCCCTAGACTTC
	MLKL-F	TCCTCTGGGAAATCGCCACTGG
	MLKL-R	GCTCCTGCTGCCGCTTCAC
	SERPING1-F	GATGCTATTCGTTGAACCCATC
	SERPING1-R	CAAGTCAGAGCAGAGAGTAACA
	TRIM21-F	TCCTTCTACAACATCACTGACC
	TRIM21-R	CAATATTCAGTGGACAGAGGGT

Note: F means forward, and R means reverse.
